# An observational study of the cause and frequency of prescription rework in community pharmacies

**DOI:** 10.1007/s11096-023-01563-3

**Published:** 2023-05-09

**Authors:** Frank Olden, Kieran Dalton

**Affiliations:** School of Pharmacy, University College Cork, College Road, Cork, Ireland

**Keywords:** Community pharmacies, Efficiency, Pharmacists, Prescriptions, Workflow, Workload

## Abstract

**Background:**

When prescriptions are being processed in pharmacies, ‘*rework*’ is a phenomenon where an activity occurs that requires the return to a prior procedural step in the process for correction. To date, little is known regarding rework prevalence in community pharmacies or how this might be minimised.

**Aim:**

To evaluate the cause and frequency of prescription rework in community pharmacies.

**Method:**

A list of reworks was designed for community pharmacists to self-record prescription rework instances and causes in their workplace across a two-week period. Community pharmacists in Ireland were recruited via convenience sampling and snowballing. Descriptive statistics were used to assess rework frequency according to the various causes, as well as the pharmacist and pharmacy characteristics.

**Results:**

Eight pharmacists participated, recording 325 reworks across 92.9% of the 65 study days (mean 5 reworks/day). The pharmacists’ mean ranged from 1.82 to 15 reworks/day. Pharmacists and pharmacy technicians alone or together were involved in 72.3% of reworks. The three most common rework categories were involving labelling errors (22.8%), prepared prescriptions which necessitated opening and repackaging (15.1%), and medication owings to patients (13.9%).

**Conclusion:**

This study reveals that prescription rework occurs frequently in community pharmacies and has provided an indication of some of the main causes. These findings demonstrate areas where pharmacy staff can address rework and should aid the development of approaches to minimise rework in future – thus decreasing workload and facilitating more time for community pharmacy staff to focus on providing patient care.

**Supplementary Information:**

The online version contains supplementary material available at 10.1007/s11096-023-01563-3.

## Impact statements

- This is the first study to evaluate the prevalence of a wide variety of prescription reworks in community pharmacies.
- This research found that at least five instances of prescription rework occur per day in community pharmacies on average, which is likely to be an underestimate.
- The list of prescription reworks developed may be useful for both pharmacy staff and researchers to assess rework prevalence in future.
- This study indicates that prescription reworks represent a significant time burden to pharmacists and add to the complexity of providing pharmaceutical care to patients.
- These findings highlight the need for strategies to be developed and implemented to minimise prescription rework in community pharmacies.

## Introduction

Pharmacists’ workload in community pharmacies is rising [1–3], which is partly attributed to increased dispensing due to patients living longer with more medical conditions, greater provision of patient services, and a growing administrative burden [4–6]. This escalating workload not only impacts patients’ access to clinical pharmacy services [7, 8], but has also been associated with pharmacist stress, burnout, reduced job satisfaction, a higher rate of job turnover, and decreased levels of pharmacists’ health and well-being [9–13].

One method of reducing this workload in community pharmacy, and perhaps consequently the risk of burnout, is through increased efficiency in procedural practice. Lean principles have been applied previously to pharmacies and other healthcare settings to improve the efficiency of clinical processes, reduce workload, and enhance the quality of healthcare [3, 14–18]. Lean principles help to create maximum value for patients by reducing waste and variation, levelling workload, and engaging staff to do work in a process [16, 19]. Waste occurs when a step fails to add value or results in redundancy to the next user, which impedes quality and flow and should be eliminated [20].

**Fig. 1:**
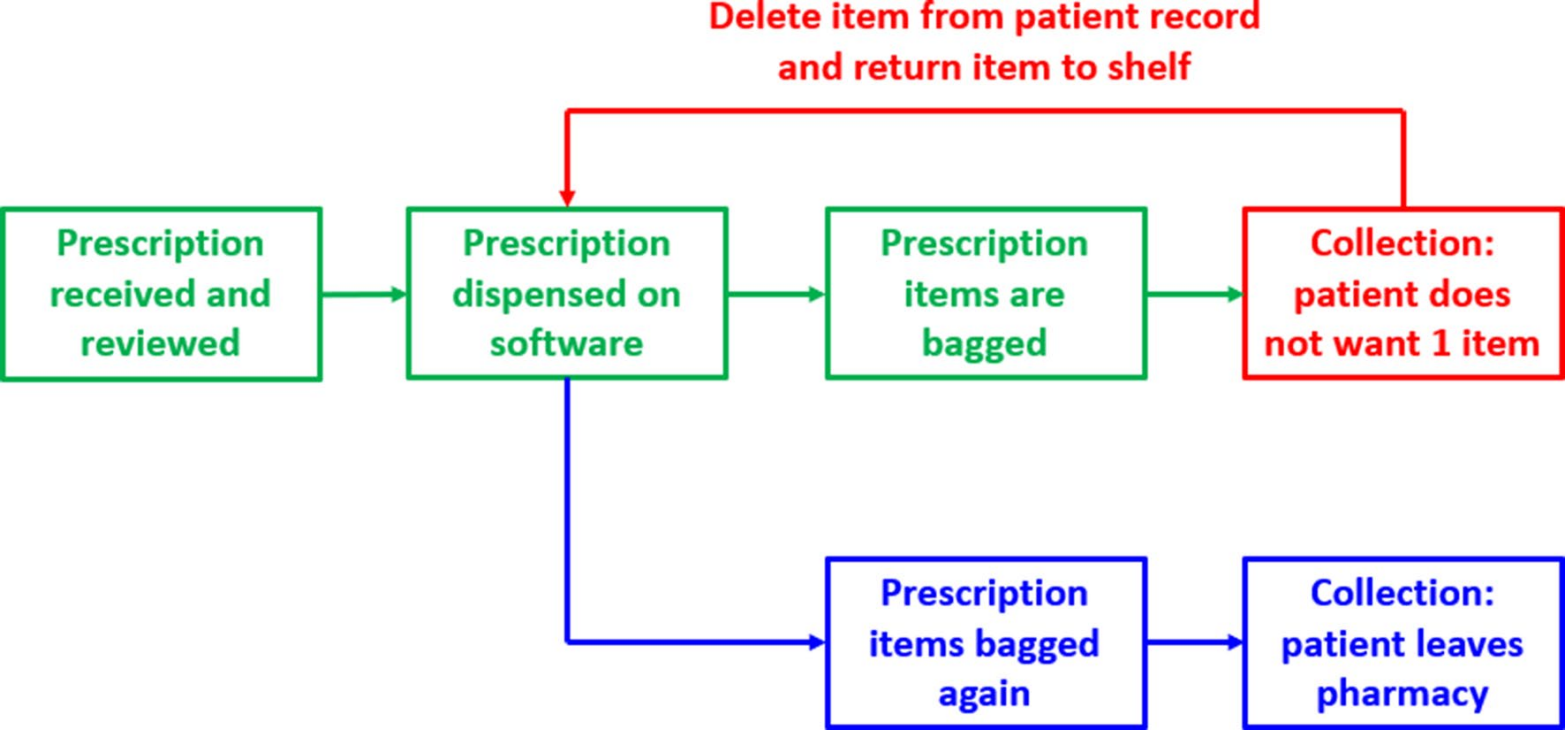
Example of a rework in prescription processing in community pharmacy. Green: normal prescription processing steps. Red: defects and corrective actions to allow return to a previous step. Blue: steps repeated as part of rework process

Within lean principles, defects and inappropriate processing are two of seven forms of waste and lead to ‘*rework*’ [17]. Whilst there is no consensus definition of rework from a community pharmacy perspective, Nickman et al*.* have defined a prescription rework as “*any activity during a medication dispensing cycle such as, but not limited to, incorrect medication or labelling errors that cause prescription processing to revert to a prior procedural step for correction*” [21]. Figure 1 shows an example of a prescription rework in community pharmacy, and how defects at any point can lead to additional work, such as corrective actions and repeating previous steps. Unsurprisingly, rework has been shown to heighten workplace stress, reduce worker morale, and increase patient frustration [22, 23]. In considering community pharmacists’ increased workloads and how this may be affecting clinical care, rework identification and strategies to minimise its occurrence should be beneficial to both pharmacy staff and patients.

Whilst errors and near misses in community pharmacies have been widely studied [24, 25], research on rework in this setting is limited [3, 21, 26, 27]. Errors may contribute to rework, but there are many more dimensions to rework – meaning that previous estimates of the impact of errors on pharmacy staff time will not reflect the true time consumed by rework. To date, some factors that contribute to rework have been identified, including workflow issues, high telephone call volume and distraction, prescriptions requiring prescriber clarification, and patients’ failure to collect automatic prescriptions refills [3, 21, 26, 27]. What is not known, however, is the frequency with which prescription rework occurs in community pharmacies and its causes. Capturing this information is important as it may help inform intervention design to minimise rework frequency for pharmacy staff, thus reducing workload and facilitating the reallocation of pharmacists’ time from reworking defects to providing clinical pharmacy services and enhancing patient care.

### Aim

To evaluate the frequency and cause of prescription rework occurring in community pharmacies.

### Ethics approval

Study approval was attained in May 2021 from the School of Pharmacy Social Research Ethics Committee, University College Cork (reference 2021-003).

## Method

The Strengthening the Reporting of Observational Studies in Epidemiology (STROBE) Statement guided reporting in this study [28].

### Development of data collection materials

A self-reporting data collection form (Supplementary Material) was created for pharmacists to record the instances and causes of rework identified in their community pharmacy workplace. The materials provided were formulated based on previous literature on rework in community pharmacy and similar data collection processes [3, 21, 26, 27], as well as the authors’ experience of working in community pharmacy. The form was piloted by two pharmacists in different community pharmacies over a three-day period. Minor revisions were made to the data collection materials after piloting and prior to ethics committee approval.

The first section of the data collection form allowed participants to record pharmacy characteristics (location, pharmacy type, staffing levels, average items dispensed per day) and pharmacist characteristics (age, gender, years post qualification, and role within the pharmacy). The second section included a table to record the date a rework occurred, the approximate time, the rework code, the person involved, and a section to gather any additional comments. To enable participants to identify rework codes, a key (Supplementary Material) was provided – detailing eight categories of rework that may occur during prescription processing, and individual rework types under these. One final category was included to allow participants document new reworks they identified which did not fall under any of the previous categories.

Instructions on how to complete the data collection form were created for participants to review (Supplementary Material). These instructions informed participants that each rework may have more than one cause; in these cases, pharmacists should record as many rework codes as required to capture all relevant rework types and causes. Participants were also reminded of the study’s aim and ‘*rework*’ definition, which was based on the Nickman et al*.* definition described above [21]*.*

### Sample size

Given the paucity of data on rework from the literature, it was not clear what would be an appropriate number of pharmacists to recruit. The initial piloting of the data collection form in March 2021 returned a mean of 15 reworks per pharmacist per day. Based on this figure and hypothesising a minimum of five reworks and a maximum of 25 reworks per day, it was estimated that over a two-week period (assuming a five-day working week), a participant would record approximately 50–250 reworks. In light of the COVID-19 pandemic, the researchers proposed that it may be feasible to recruit 10 pharmacists for data collection, which would provide approximately 500–2500 reworks for analysis. To account for variability between pharmacies, the study aimed to include a minimum of two of each of the pharmacy types – i.e. two independent pharmacies, two small chain pharmacies (< 10 pharmacies/chain), and two large chain pharmacies (≥ 10 pharmacies/chain).

### Participant recruitment

Community pharmacists registered with the Pharmaceutical Society of Ireland who were in full-time employment in a single community pharmacy in Ireland were eligible for inclusion. Pharmacists working in the same pharmacy as a participating pharmacist were not eligible to participate. Pharmacists known to the primary researcher were recruited by email or telephone (convenience sampling). Thereafter, recruited participants were asked if they were aware of other eligible pharmacists who may be interested in participating (snowballing) [29]. Ten pharmacists who were interested in participating were sent an invitation email along with an information sheet and consent form. Once consent was obtained, pharmacists who agreed to participate were provided with the data collection materials and instructed to collect data over a two-week period on the days they worked.

### Data collection

Data were collected by pharmacists between June and August 2021. Each participant was the only staff member aware that data collection was taking place to minimise the possibility of the Hawthorne effect [30]. Data collection forms were returned to the primary researcher once participants completed collecting data.

### Data analysis

The data collected were entered into Microsoft® Excel. The comments section provided by the participants were screened to ensure that each rework was correctly categorised by the participant under its appropriate rework code. ‘*Other*’ reworks considered similar in nature were categorised or named by the research team according to their various causes (e.g. pricing error). Thereafter, the total number of days of data collection was tallied. Descriptive statistics were calculated, e.g. including the frequency that each individual rework type occurred. Reworks occurring per day per participant were then extracted and tabulated alongside the pharmacy and pharmacist characteristics. Descriptive statistics were then used to describe how these characteristics aligned to the rework frequency (i.e. mean, standard deviation [SD], range).

The frequency of rework was categorised according to (i) the time at which rework occurred, and (ii) the person or people involved in the rework (e.g. pharmacist, pharmacy technician, patient, prescriber). Thereafter, the percentage frequency of rework was calculated for each of the time ranges and for the groups involved.

## Results

Ten pharmacists were invited to participate, of which eight returned data within the data collection period. Two pharmacists failed to respond to the study invitation after initial contact and did not engage in follow-up emails thereafter.

### Reworks recorded

In total, participants recorded 325 reworks across 65 days (mean 5 per day; SD 4.8; range 1.8–15). Rework was recorded on 92.9% of the pharmacists’ working days. Table 1 lists the frequency of each rework type under each of the rework categories. All 32 rework types provided in the data collection form were documented to have occurred at least once throughout the study, with ‘*other*’ reworks accounting for only 8.3% of total reworks (i.e. those which occurred which did not fall under any of the pre-specified categories).

**Table 1:** Rework frequency recorded by participants

Rework categories and types	Rework frequency	% of total reworks
**1. Rework due to a prepared prescription being returned to stock**	**29**	**8.9%**
Healthmail prescription prepared and not collected	15	4.6%
Prescription duplicated	5	1.5%
Prescription was ordered by patient but not collected	3	0.9%
Prescription made up but patient considered medication too expensive	3	0.9%
Incorrect medication prepared	3	0.9%
**2. Rework on prepared prescription opened and repacked**	**49**	**15.1%**
An item was omitted from bag	24	7.4%
There was an additional unwanted item in bag	16	4.9%
A new prescription was received with changes to medication	9	2.8%
**3. Rework on phone: > 1 phone call to complete a single prescription order**	**28**	**8.6%**
Pharmacy rang patient again to reconfirm order	12	3.7%
Poor communication between staff member and patient resulted in a subsequent phone call	8	2.5%
Prescription ordered from GP, not received and patient rang to check for prescription again	7	2.2%
Patient rang again to check if prescription was ready after placing order	1	0.3%
**4. Rework due to a labelling error (correct drug, wrong label)**	**74**	**22.8%**
Wrong instructions	26	8%
Wrong quantity	17	5.2%
Wrong strength	11	3.4%
Wrong pharmaceutical form	6	1.9%
Label unclear	6	1.9%
Wrong patient	4	1.2%
Wrong brand	4	1.2%
**5. Rework due to filling errors (correct label)**	**33**	**10.2%**
Wrong quantity	13	4%
Wrong drug and/or strength	11	3.4%
Wrong brand	5	1.5%
Wrong pharmaceutical form	4	1.2%
**6. Rework due to owings**	**45**	**13.9%**
Insufficient supply of medication on shelf	32	9.9%
Item short from wholesaler	10	3.1%
Patient has a preferred brand no longer routinely stocked	2	0.6%
Owing made as originator brand not in stock (Do Not Substitute written on prescription)	1	0.3%
**7. Rework to medication update on prescription**	**14**	**4.3%**
Medication started and was added to prepared prescription	9	2.8%
A medication was stopped and required removal from a prepared prescription	5	1.5%
**8. Prescription rework due to pharmacist intervention**	**26**	**8%**
Prescriber contacted to amend dose on prescription	12	3.7%
Prescriber contacted due to illegible prescription	10	3.1%
Prescriber contacted due to drug interaction	4	1.2%
**9. Other (i.e. new reworks identified)**	**27**	**8.3%**
Pricing error	8	2.5%
Prescription not valid to meet patient’s request	4	1.2%
Prescription not due	4	1.2%
Storage error	2	0.6%
Information technology claiming issue	2	0.6%
Not stated	2	0.6%
Split information across more than one label	1	0.3%
Medication at wrong time in blister pack	1	0.3%
Medication not ready despite patient calling	1	0.3%
Patient presented with a prescription emailed to patient	1	0.3%
Rechecking for an emailed prescription	1	0.3%

The three most frequent types of individual reworks were those due to insufficient supply of medication on shelf (9.9%), wrong instructions on the label (8%), and an item being omitted from a patient’s bag (7.4%). The three most frequent rework categories were those due to labelling errors (22.8%), prepared prescriptions which required opening and repackaging (15.1%), and medication owed to patient (13.9%).

Table 2 presents the characteristics of the pharmacist participants and the pharmacy they worked in when collecting data. Table 3 details the rework frequency according to each pharmacy and pharmacist characteristic. Table 4 shows the main people that were at least partly involved in the rework and the most frequent rework types per group; the full breakdown of people involved is shown according to frequency in the Supplementary Material.

**Table 2:** Pharmacy and pharmacist characteristics

Pharmacy location	Pharmacy type	Pharmacists on duty/day	Technicians on duty/day	OTC staff on duty/day	Mean items dispensed/day	Opening hours	Role	Years post qualification	Age range	Gender	Mean reworks/day
Rural/semi-rural	Independent	1	1	1	175	9 am–7 pm	Support pharmacist^†^	30	50–59	Female	15
Suburban/large urban area	Independent	1	2	2	270	9 am–9 pm	Support pharmacist^†^	4	< 30	Male	10.7
Suburban/large urban area	Independent	1	2	2	260	9 am–6 pm	Support pharmacist^†^	4	30–39	Male	5.5
Suburban/large urban area	Large chain (≥ 10 pharmacies)	1	1	0	114	9 am–6 pm	Supervising pharmacist^*^	4	< 30	Female	3.1
Suburban/large urban area	Large Chain (≥ 10 pharmacies)	2	2	3	230	9 am–7 pm	Supervising pharmacist^*^	6	30–39	Female	3.1
Rural/semi-rural	Independent	2	2	2	250	9 am–6 pm	Support pharmacist^†^	4	< 30	Male	3
Suburban/large urban area	Small Chain (< 10 pharmacies)	2	3	2	370	9 am–7 pm	Supervising pharmacist^*^	3	< 30	Male	2.1
Rural/semi-rural	Independent	1	2	3	290	9 am–6 pm	Support pharmacist^†^	4	30–39	Female	1.8

*The supervising pharmacist is the person responsible for the day-to-day management and operation of the pharmacy. A supervising pharmacist can only act in respect of one pharmacy premises and must have a minimum of three years' post-registration experience. [40]

†A support pharmacist works under the supervising pharmacist and may be responsible for the safe and effective running of the pharmacy in the supervising pharmacist's absence

**Table 3:** Pharmacy and pharmacist characteristics and rework frequency

Characteristic		Rework frequency	
		Mean ± SD	Range
**Pharmacy location**	Suburban/large urban (*n* = 5)	4.9 ± 3.5	2.1–10.7
	Rural/semi-rural (*n* = 3)	6.6 ± 7.3	1.8–15
**Pharmacy type**	Independent pharmacy (*n* = 5)	7.2 ± 5.5	1.8–15
	Large chain (*n* = 2)	3.1	–
	Small chain (*n* = 1)	2.1	–
**Staffing**	1 pharmacist and 2 dispensary staff (*n* = 2)	9.1	3.1–15
	1 pharmacist and 3 dispensary staff (*n* = 3)	6 ± 4.4	1.8–10.7
	2 pharmacists and ≥ 4 dispensary staff (*n* = 3)	2.7 ± 0.6	2.1–3.1
**Pharmacist role**	Supervising pharmacist* (*n* = 3)	2.8 ± 0.6	2.1–3.1
	Support pharmacist^†^ (*n* = 5)	7.2 ± 5.5	1.8–15
**Years post qualification**	3 Years (*n* = 1)	2.1	–
	4 Years (*n* = 5)	4.8 ± 3.5	1.8–10.7
	6 Years (*n* = 1)	3.1	–
	30 Years (*n* = 1)	15	–
**Age**	< 30 (*n* = 4)	5.6 ± 4	2.1–10.7
	30–40 (*n* = 3)	3.5 ± 1.9	1.8–5.5
	50–59 (*n* = 1)	15	–
**Gender**	Male (*n* = 4)	5.3 ± 3.9	2.1–10.7
	Female (*n* = 4)	5.8 ± 3.9	1.8–15

**Pharmacy opening hours and approximate time of rework**

Pharmacies open 9 am-6 pm (*n* = 4) and 9 am-7 pm (*n* = 3): the highest percentile of reworks were recorded in the morning (9 am-12 pm: 36.5% and 37.2% respectively), followed by the late afternoon (3 pm-6 pm; 34.6% and 30.1% respectively), followed by the afternoon (12 pm-3 pm; 29% and 28.9% respectively). In pharmacies open 9 am-7 pm, 3.9% of reworks occurred from 6 pm-7 pm

For the sole pharmacy open from 9 am-9 pm, the highest percentile of reworks was recorded after 6 pm (37.5%), followed by from 12 pm-3 pm (21.9%), with 20.3% each occurring from 9 am-12 pm and 3 pm-6 pm (20.3% each)

^*^The supervising pharmacist is the person responsible for the day-to-day management and operation of the pharmacy. A supervising pharmacist can only act in respect of one pharmacy premises and must have a minimum of three years' post-registration experience [40]

^†^A support pharmacist works under the supervising pharmacist and may be responsible for the safe and effective running of the pharmacy in the supervising pharmacist's absence.

SD: Standard deviation

**Table 4:** Main people involved in reworks

People involved and most frequent rework types	Number of reworks at least partly involved in (% total)
**Pharmacists** Pharmacists alone were involved in 33.5% of reworks (*n* = 109), the most frequent of which were insufficient supply of medication on shelf (*n* = 15), ‘*other*’ reworks (*n* = 12), and an item being omitted from a bag (*n* = 10).	227 (69.8%)
**Pharmacy technicians** Technicians alone were involved in 22.5% of reworks. The three most frequent reworks technicians were involved in included insufficient supply of medication on shelf (*n* = 8), wrong quantity of medication prepared (*n* = 8), and wrong drug and/or strength (*n* = 8). Overall, pharmacists and technicians alone or together were involved in 72.3% of reworks (*n* = 235). Both pharmacists and technicians together were involved in 16.3% of reworks; the most frequent reworks here included the prescription sent via electronic prescription transfer being prepared and not collected (*n* = 12), wrong instructions on label (*n* = 11), and wrong strength on label (*n* = 6).	144 (44.3%)
**Patients** Patients and pharmacists together were involved in 10.2% of reworks, the most frequent of which were having an additional unwanted item in the bag (*n* = 5), insufficient supply of medication on shelf (*n* = 4), and the pharmacy contacting the patient by phone again to confirm order (*n* = 4).	64 (19.7%)
**Prescribers** The prescriber and pharmacist were involved in 4% of reworks; the most frequent reworks included contacting the prescriber to amend dose on the prescription (*n* = 5) and contacting the prescriber due to an illegible prescription (*n* = 3).	25 (7.7%)

### Additional comments on reworks provided by pharmacists

All participants provided comments on some of the reworks, which allowed for explanations and nuances to be identified for some rework types. Comments included reworks involving high-risk medications, such as oral chemotherapy, immunosuppressants, hypnotics, insulin-containing products, non-steroidal anti-inflammatory drugs, and antibiotics.

Other examples of factors that contributed to rework included:

- Items which required refrigeration being left out of the refrigerator.
- Prescriptions with the duration of treatment omitted.
- Incomplete controlled drug prescription requirements.
- Instructions for pravastatin to be changed from daytime to night-time dosing.
- Typographical errors on labels.
- A blister pack that required a change in the time of day that the dose was packaged for administration.

## Discussion

### Statement of key findings

This is the first study to investigate the prevalence of a wide variety of prescription reworks in community pharmacies. The results indicate that at least five instances of rework occur per day on average in community pharmacies, with rework most commonly caused by labelling errors (22.8%). Most rework was caused internally by staff, with pharmacists and pharmacy technicians alone involved in 72.3% of all reworks. Rework on prepared prescriptions occurred frequently in this study; an item being omitted from a patient’s bag was the third most frequent rework recorded, while the addition of unwanted medication in the patient’s bag was the fifth most frequent type. These results therefore build on existing evidence regarding the importance of communication between pharmacy staff and patients in preventing rework [3].

### Strengths and limitations

This study’s small sample size limited the ability to robustly explore how pharmacist or pharmacy characteristics may have impacted on rework frequency. Pharmacists being too busy may have affected recruitment, especially as data collection was conducted during the COVID-19 pandemic – where pharmacists were busier undertaking additional duties (e.g. vaccinating against COVID-19) [31, 32]. Furthermore, this busyness informed the sampling approach used and may have been a reason that not all pharmacists provided a full two-week dataset.

The rework list developed for this study is a strength, as its pre-specified categories captured 91.7% of reworks. Moreover, its relevance to practice is indicated by the fact that at least one of each pre-specified rework type was captured, even with a small sample size. This study is strengthened further by the pharmacists’ discreet rework recording, which minimised the Hawthorne effect on other pharmacy staff [30]. However, it is likely that the prevalence rate reported was an underestimate as some reworks caused by other staff members likely did not reach pharmacist awareness. Furthermore, the self-reporting nature means that rework recording could have been reduced further by memory lapses and social desirability bias [33]. Independent observation may provide a more accurate insight into rework occurrence in prescription processing; however, it too has limitations, e.g. harder to minimise the Hawthorne effect unless done discreetly, such as through video recording. Furthermore, self-reporting would likely be required to capture nearly all rework instances, as it would be challenging for an independent observer to be aware of all such instances.

### Interpretation and implications for future research and practice

This study found that insufficient medication on the shelf was the most frequent individual cause of rework (9.9%), and was responsible for 71.1% of rework related to owing medication. Partial medication supply due to owings may contribute to lack of medication concordance and can have serious implications for patients when the full treatment course is not completed (e.g. for antimicrobials). Furthermore, having insufficient medication on the shelf can result in significant prescription rework, whereby a cascade of processes is triggered. For example: if the dispensing label has been generated prior to spotting the insufficiency, this label may require amendment on the patient medication record (PMR), an order must be placed with the wholesaler, and reassurance provided to the patient for a time when the medication will be restocked. Once the medication becomes available, the PMR must be updated to generate a label before being checked and provided to the patient. This example highlights the complexity that rework can propagate in a community pharmacy. While such medication owings may be due to external factors beyond pharmacists’ control, such as medication shortages from the wholesaler, another important cause is poor inventory management. Given the high prevalence of medication insufficiency found here, this highlights that community pharmacies should consider their current inventory management system’s effectiveness to help minimise such unnecessary rework cascades that add significantly to staff workload.

Approximately 5% of reworks in this study were due to prescriptions being prepared and not collected after they had been sent via Healthmail (a national secure email system). This is interesting as electronic prescription transfer to pharmacies via Healthmail was only newly permitted since April 2020 in Ireland. Whilst preparing prescriptions in advance may contribute positively by smoothing out staff workload demands, it may also contribute negatively due to increased rework when prepared prescriptions are not collected [34], as shown previously in a study with automatic prescription dispensing [27]. It can take time for new technologies to integrate into prescription processing; therefore, community pharmacies should aim to ensure standard operating procedures are in place to minimise rework and workflow disturbances when such new technologies are introduced.

This study considered rework frequency in relation to the time of day. Rework occurred more frequently in the morning (9 am to 12 pm) and the late afternoon (3 pm to 6 pm). This may be due to higher prescription volume at these times, with lunchtime schedules impacting volume in the intervening interval. However, the sole pharmacist working from 9 am to 9 pm recorded the majority of rework in the evening after 6 pm. Therefore, fatigue may have been a contributory factor to increased rework frequency, especially as its negative effect on performance is well documented [35–37]. Many variables such as prescription volume, opening hours, fatigue, and workload warrant further investigation as to their role in affecting pharmacy rework.

This study provides an indicator that staffing levels and the pharmacist role may impact rework. Pharmacies with higher levels of dispensary staff (2 pharmacists and total dispensary staff number ≥ 4) recorded less rework than pharmacies with lower levels of dispensary staff (1 pharmacist and total dispensary staff number ≤ 3), regardless of pharmacy type. However, while the generalisability of the results are limited by the small sample size, they are in line with previous research that indicates that inadequate pharmacist cover leads to medication errors [38]. Although rework and medication errors have distinct definitions, both are failures to complete a planned action as intended [27, 39]. Additionally, this study found a lower average of reworks recorded by supervising pharmacists (responsible for the day-to-day management and operation of the pharmacy) [40] compared to support pharmacists. This could be due to supervising pharmacists being less involved with prescription processing due to additional responsibilities (e.g. administrative tasks). Under-reporting by supervising pharmacists, due to social desirability bias regarding a greater reputation to uphold, may also be a possible factor to reduced rework documentation. However, it is also important to note that pharmacists with a greater ability to detect medication-related problems may contribute to increased rework (e.g. through interventions) [41], but ultimately this is for patients’ benefit. Therefore, this perhaps emphasises the value in differentiating between necessary rework and unnecessary rework. Future studies investigating rework should have a larger sample size to better explore these factors and facilitate greater generalisability across the sector.

Given the usefulness of this study’s rework list, future researchers may wish to use this to guide data collection, with adaptation where needed to develop a more comprehensive list incorporating some of the ‘*other*’ rework types identified, as well as factoring in local rework contributors. Pharmacy staff may also find this list useful to audit the prevalence in their setting, which may help identify staff training needs (e.g. technician making prescription filling errors), thus contributing to a more positive safety culture. Future qualitative work is also needed to develop strategies to minimise rework in community pharmacies. It has been previously calculated that an average community pharmacy spends four hours of pharmacist time per week reworking prescriptions (i.e. 208 h per pharmacy per year) [42]; therefore, minimising rework could have significant time and cost benefits, and future researchers should consider evaluating the cost-effectiveness of strategies targeted at reducing rework.

## Conclusion

This study has shown that rework happens regularly in community pharmacies, and has provided an insight into the type of rework that occurs. The most common causes of rework were due to labelling errors, owings (mostly due to insufficient stock), and prepared prescriptions which required opening and repackaging (due to omissions or unnecessary additions). While pharmacy and pharmacist characteristics may have influenced the reported rework frequency, it was not possible to conclusively establish an association between these due to the small sample size. Given that the rework list designed for this study was successful in capturing over 91.7% of rework instances with the pre-specified rework types, it would therefore be recommended that this list is used to help inform studies with a larger sample size investigating rework in community pharmacies in future. Importantly, this research is valuable as it generates awareness about rework to pharmacy staff and researchers, making them consider how this rework can be minimised, thus reducing workload, and ultimately facilitating time reallocation to enhance patient safety during prescription processing and the provision of clinical services to meet patients’ needs.

## Supplementary Information

Below is the link to the electronic supplementary material.

Supplementary file1 (DOCX 56 KB)
