# Supplementary material for: An observational study of the cause and frequency of prescription rework in community pharmacies
Source: Int J Clin Pharm. 2023 May 9;45(4):903–12. doi: 10.1007/s11096-023-01563-3 (PMC10169171; doi:10.1007/s11096-023-01563-3)
Supplement: Supplementary file 1 — Supplementary file1 (DOCX 56 KB) [file 11096_2023_1563_MOESM1_ESM.docx]

## Appendix 1: Data collection form

***Pharmacy Characteristics***

1. Please tick the box which best describes your pharmacy location:

City centre.

Suburban/large urban area (other than city centre).

Rural/semi-rural.

1. Please select from the list which type of pharmacy you work in:

An ‘independent pharmacy’ (i.e. a single pharmacy that trades on its own).

A pharmacy which is part of a small chain (Less than 10 pharmacies)

A pharmacy which is part of a large chain (10 or more pharmacies)

1. For each of the listed staff members below, calculate the average number of each type that would be present in the pharmacy on any given day:

| **Type of staff member** | **Average number of staff per day** |
| --- | --- |
| Pharmacists |  |
| Dispensary staff (other than the pharmacist) |  |
| OTC staff |  |

1. In the box below, please provide the average number of items dispensed per day in the pharmacy you work in.

*Note: to get the average number of items dispensed per day, use the pharmacy dispensary software to retrieve the total number of items dispensed in one month and divide this figure by the number of days the pharmacy was open to trade in that month.*

***Pharmacist Characteristics***

1. Select the community pharmacist role(s) that currently apply to you. Tick all that apply:

Pharmacy Owner

Superintendent Pharmacist

Supervising Pharmacist

Support Pharmacist

Other(s)

1. In the box below, please provide the number of years of post-qualification experience you have completed as a pharmacist.

*For ease of record, please round the number of years down to the nearest whole number.*

1. Please select your age range:

Aged 29 or less

Aged 30 - 39

Aged 40 - 49

Aged 50 - 59

Aged 60 or more

1. What is your gender?

***Rework data collection tool.***

| **Date** | **Approximate Time** | **Rework Code(s)** | **Person or People involved *(e.g. Pharmacist / Technician / Patient / Doctor etc.)*** | **Comments** |
| --- | --- | --- | --- | --- |
|  |  |  |  |  |
|  |  |  |  |  |
|  |  |  |  |  |
|  |  |  |  |  |
|  |  |  |  |  |
|  |  |  |  |  |
|  |  |  |  |  |
|  |  |  |  |  |
|  |  |  |  |  |
|  |  |  |  |  |
|  |  |  |  |  |
|  |  |  |  |  |
|  |  |  |  |  |
|  |  |  |  |  |
|  |  |  |  |  |
|  |  |  |  |  |
|  |  |  |  |  |
|  |  |  |  |  |
|  |  |  |  |  |

## Appendix 2: Key detailing types of rework and cause.

| **1. Rework due to a prepared prescription being returned to stock** |  |
| --- | --- |
| **1a.** Prescription duplicated |  |
| **1b.** Healthmail prescription prepared and not collected |  |
| **1c.** Prescription was ordered by patient but not collected |  |
| **1d.** Prescription made up but patient considered medication too expensive |  |
| **1e.** Incorrect medication prepared |  |
| **2. Rework on prepared prescription - opened and repacked** |  |
| **2a**. There was an additional unwanted item in bag |  |
| **2b**. An item was omitted from bag |  |
| **2c.** A new prescription was received with changes to medication |  |
| **3. Rework on phone: >1 phone call to complete a single prescription order** |  |
| **3a.** Patient rang again to check if prescription was ready after placing order |  |
| **3b.** Pharmacy rang patient again to reconfirm order |  |
| **3c.** Prescription ordered from GP, not received and patient rang to check for prescription again |  |
| **3d.** Poor communication between staff member and patient resulted in a subsequent phone call |  |
| **4. Rework due to labelling error (correct drug, wrong label)** |  |
| **4a.** Wrong strength |  |
| **4b.** Wrong patient |  |
| **4c.** Wrong instructions |  |
| **4d.** Wrong brand |  |
| **4e.** Wrong pharmaceutical form |  |
| **4f.** Wrong quantity |  |
| **4g.** Label unclear |  |
| **5. Rework due to filling errors (correct label)** |  |
| **5a**. Wrong drug and/or strength |  |
| **5b.** Wrong brand |  |
| **5c.** Wrong pharmaceutical form |  |
| **5d.** Wrong quantity |  |
| **6. Rework due to owings** |  |
| **6a.** Insufficient supply of medication on shelf |  |
| **6b.** Patient has a preferred brand no longer routinely stocked |  |
| **6c.** Owing made as originator brand not in stock (‘Do Not Substitute’ written on prescription) |  |
| **6d.** Item short from wholesaler |  |
| **7. Rework due to a medication update on prescription** |  |
| **7a.** A medication was stopped and required removal from a prepared prescription |  |
| **7b.** Medication started and was added to prepared prescription |  |
| **8. Prescription rework due to pharmacist intervention** |  |
| **8a.** Prescriber contacted to amend dose on prescription |  |
| **8b.** Prescriber contacted due to illegible prescription |  |
| **8c.** Prescriber contacted due to drug interaction |  |
| **9. New rework identified** |  |
| **9a.** Other |  |

##

## Appendix 3: Instructions to complete data collection form

1. Please allocate a period of two weeks to input data into the rework data collection tool. As the study participant, only record reworks on days you work over the two-week period.
2. The Hawthorne effect is a change in behaviour that occurs in response to a person’s awareness of being observed. In order to minimise the Hawthorne effect, the rework data collection tool should be completed without other staff members being aware of the study being completed. Therefore, exercise caution when recording reworks.

Note if you are not the pharmacy owner/superintendent pharmacist: although the pharmacy owner/superintendent pharmacist has given permission for this study to take place, please ensure they do not know exactly when the study is to take place and that they do not inform any other staff members about the study.

1. Use Table 1 as a reference to categorise reworks as they occur. There are 8 types of reworks listed. Each of the eight categories have subcategories for the cause of each rework. In essence, each rework listed has a corresponding numerical and alphabetical “rework code”, e.g. 1a, 3c etc.
2. To complete the rework data collection tool (Table 2), continue to work as normal. As reworks arise throughout the working day, record information regarding the rework under the headings provided in Table 2 – Date, Approximate time, rework code, person or people involved, and any additional comments that may help explain a rework.
3. Each rework may have more than one cause. In these cases, record as many rework codes as required.
4. Note: To maintain confidentiality, do not record identifiable information of patients (or their carers/advocates), staff, or other healthcare professionals. The staff title of a person involved in a rework may be recorded where appropriate: e.g. OTC assistant, technician, pharmacist, doctor.
5. If a new rework occurs and it is not listed in the key, the rework can be recorded using the rework code “9a” (other). Provide a brief description of the rework in the comment area of Table 2.

**Appendix 4: People involved in rework according to frequency**

| **Person or People involved** | **Number of reworks** | **% of total reworks** |
| --- | --- | --- |
| Pharmacist only | 109 | 33.5% |
| Technician only | 73 | 22.5% |
| Pharmacist and Technician | 53 | 16.3% |
| Pharmacist and Patient | 33 | 10.2% |
| Pharmacist and Prescriber | 13 | 4% |
| Patient only | 11 | 3.4% |
| Pharmacist, Technician, and Patient | 9 | 2.8% |
| Patient and Technician | 4 | 1.2% |
| Pharmacist, Patient, and Prescriber | 4 | 1.2% |
| Patient and Prescriber | 3 | 0.9% |
| Pharmacist and Carer | 3 | 0.9% |
| Pharmacist, Technician, and Prescriber | 2 | 0.9% |
| Technician and Prescriber | 2 | 0.6% |
| Pharmacy Student | 2 | 0.6% |
| Prescriber only | 1 | 0.3% |
| Technician and Carer | 1 | 0.3% |
| OTC staff only | 1 | 0.3% |
| Pharmacist and OTC staff | 1 | 0.3% |
